# GC-MS metabolomics reveals metabolic differences of the farmed Mandarin fish *Siniperca chuatsi* in recirculating ponds aquaculture system and pond

**DOI:** 10.1038/s41598-020-63252-9

**Published:** 2020-04-08

**Authors:** Mingsong Xiao, Kelin Qian, Yuliang Wang, Fangyin Bao

**Affiliations:** 1 College of Life and Health Science, Anhui Science and Technology University, Fengyang, 233100 China; 2 Chuzhou Nanqiao District Yangtze River Aquaculture Breeding Ground, Chuzhou, 239000 China

**Keywords:** Metabolomics, Metabolism

## Abstract

*Siniperca chuatsi* is currently one of the most important economic farmed freshwater fish in China. The aim of this study was to evaluate the metabolic profile of recirculating ponds aquaculture system (RAS)-farmed *S. chuatsi*. Gas Chromatography-Mass Spectrophotometry (GC-MS) metabolomic platform was used to comprehensively analyze the effects of recirculating ponds aquaculture system (RAS) on the Mandarin fish *S. chuatsi* metabolism. Database searching and statistical analysis revealed that there were altogether 335 metabolites quantified (similarity > 0) and 205 metabolites were identified by mass spectrum matching with a spectral similarity > 700. Among the 335 metabolites quantified, 33 metabolites were significantly different (VIP > 1 and p < 0.05) between RAS and pond groups. In these thirty-three metabolites, taurine, 1-Hexadecanol, Shikimic Acid, Alloxanoic Acid and Acetaminophen were higher in the pond group, while 28 metabolites were increased notably in the RAS group. The biosynthesis of unsaturated fatty acids, lysosome, tryptophan metabolism were recommended as the KEGG pathway maps for *S. chuatsi* farmed in RAS. RAS can provide comprehensive benefits to the effects of *Siniperca chuatsi* metabolism, which suggest RAS is an efficient, economic, and environmentally friendly farming system compared to pond system.

## Introduction

The Chinese mandarin fish *Siniperca chuatsi* (Basilewsky) is a freshwater fish with high economic value and is endemic to East Asia, specially distributed in the Yangtze River drainage in China^1^. The resources of wild *S. chuatsi* have declined dramatically because of water pollution, damming and over-fishing in recent years^2^. At present, with the social and economic development, the popularity of the fish has increased, the catches from the wild could not meet the demand, and the resulting market price has created much interest in the aquaculture of *S. chuatsi*. In order to adapt to market demand, the stocking and culture techniques were developed for the *S. chuatsi*. The mandarin fish is widely cultured throughout the country, and is also important in stocking fisheries in lakes and reservoirs^3^. However, outbreaks of the diseases caused by viruses, bacteria and parasites have brought severe economic losses to the *S. chuatsi* breeding industry^4,5^. Due to water pollution, low survival rate and the nutrients issue of a serious waste of water, traditional ponds of *S. chuatsi* at this stage have encountered many difficulties, which have largely hindered its commercial exploitation. Recirculating ponds aquaculture system (RAS) is a new ecological ponds aquaculture guided by the idea of cyclic economy^6^. RAS have gained increasing interest in recent years as a means to intensify fish production while at the same time reduce water and land usage, minimizing the adverse environmental impact^7,8^. The pond healthy breeding technology demonstration and promotion of *Siniperca chuatsi* was carried out to meet the needs of high-quality aquatic products and promote the aquaculture industry in restructuring.

Metabolomics is an “omics” technique that is situated downstream of proteomics, transcriptomics and genomics^9^. Metabolomics is defined as the quantitative measurement of the dynamic multiparametric metabolic response of living systems to pathophysiological stimuli or genetic modification^10^, which has been proposed as a powerful tool for exploring the complex relationship between nutrition and health in nutrition research^11–13^. Gas chromatography-mass spectrometry (GC-MS) has many merits, for example powerful resolving capability and high reproducibility, that render it extensively useful in the field of metabolomic profiling^14^. Serum metabonomic profiles of RAS- and pond-cultured *S. chuatsi* were detected using GC-MS techniques employed to globally characterize changes. So far, this is the first time that GC-MS techniques were applied to identify the metabolic profile of the RAS- and pond-cultured *S. chuatsi*.

## Results

### Metabolomic profiling through GC- MS

**Figure 1:**
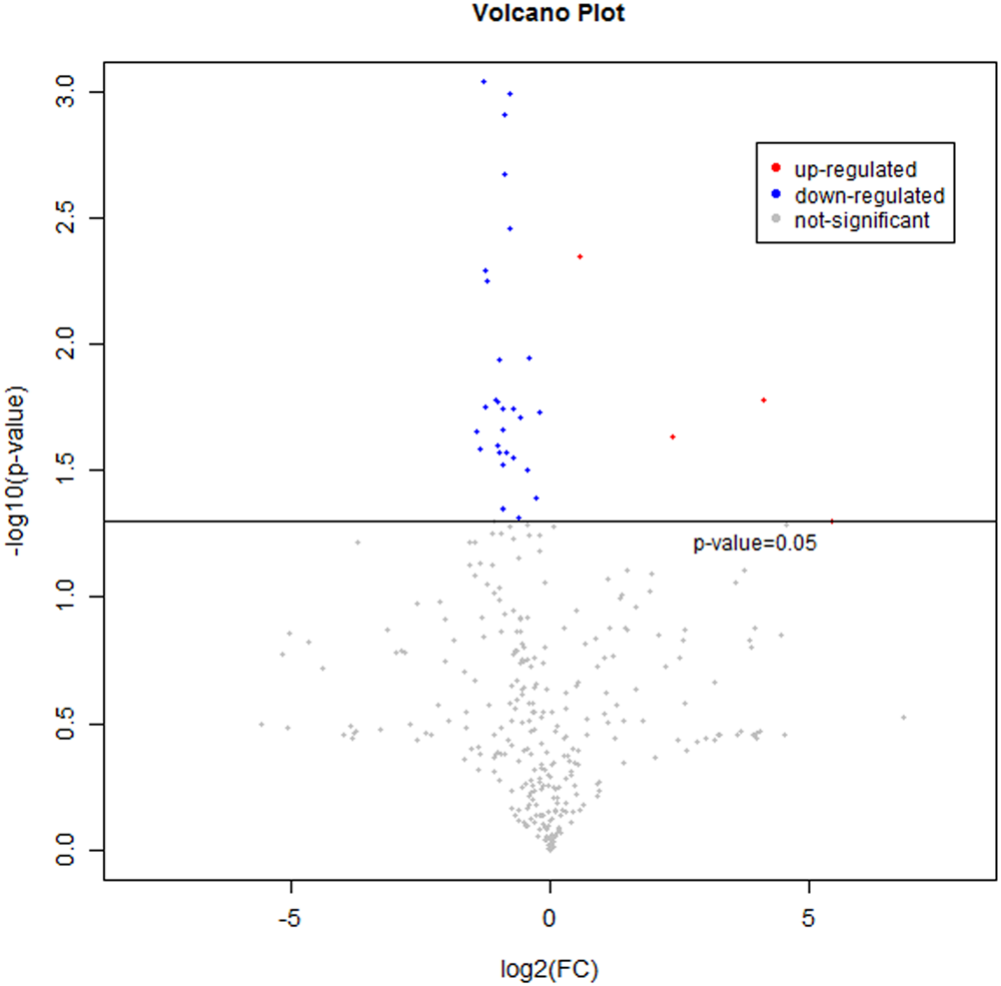
Scatter plots of metabolomics between the RAS group and pond group. The horizontal and vertical axes indicate expression levels of the metabolites in the two groups. The red and blue dots represent up- and down-regulated metabolites with significant differential metabolites (−log^10^ (p value) and log^2^ (FC), P value < 0.05) in *Siniperca chuatsi*, respectively. The gray dots represent the metabolites without significant differential metabolites.

A total of 775 valid peaks were identified in the serum. 335 peaks were retained after filtering and de-noising, and most peaks were identified and attributed to endogenous metabolites (similarity > 0) (Table S1). Among these, 205 metabolites based on mass spectrum matching were identified (spectral similarity value > 700), which indicated high credibility (Table S1). The specific variable quantities were indicated in the RAS compared with the pond by fold-change (FC) value. The metabolites of RAS- and pond- farmed *S. chuatsi* could be visually divided into down-regulation (FC value < 1) and up-regulation (FC value > 1). As shown in Fig. 1, metabolites down-regulation was more common than up-regulation at RAS- and pond- farmed *S. chuatsi* groups. Compared with the RAS group, only 3 metabolites were upregulated in the pond group. 33 metabolites were significantly different (p < 0.05 and VIP > 1) between pond and RAS groups in the total 335 metabolites quantified (Table 1), which consisted of 19 metabolites with similarity > 700 and 14 metabolites with similarity <700. In the 19 metabolites with similarity >700, inositol-4-monophosphate, mannonic acid, linoleic acid, isohexonic acid, 2-hydroxybutanoic acid, 2-deoxytetronic acid, uric acid, 2-hydroxyhexanoic acid, 1-monopalmitin, behenic acid, isoheptadecanoic acid, diglycerol, mannose-6-phosphate, 2-ketoisovaleric acid, lauric acid, arachidic acid and xanthurenic acid were higher in the RAS group, while taurine and 1-hexadecanol were higher in the pond group.

**Table 1:** Identification of significantly different metabolites in serum between the RAS and Pond groups.

Metabolite name	Similarity	Average RT (min)	VIP	*p-*value	log_2_(FC)	RAS group	Pond group
Inositol-4-Monophosphate	956	30.91	2.077	0.001	−0.776	0.2269	0.1325
Mannonic Acid	918	25.40	1.613	0.031	−0.444	0.1943	0.1429
Linoleic Acid	910	28.81	1.725	0.025	−1.002	0.0883	0.0441
Taurine	890	18.80	1.700	0.023	2.359	0.0956	0.4903
Isohexonic Acid	875	25.80	1.639	0.049	−0.600	0.0042	0.0028
2-Hydroxybutanoic Acid	869	7.89	2.006	0.001	−0.865	0.0342	0.0188
2-Deoxytetronic Acid	867	12.06	1.956	0.012	−0.976	0.0053	0.0027
Uric Acid	845	27.15	1.678	0.028	−0.706	0.0014	0.0009
2-Hydroxyhexanoic Acid	841	9.76	2.035	0.001	−1.282	0.0141	0.0058
1-Monopalmitin	830	32.47	1.674	0.027	−0.851	0.0867	0.0481
Behenic Acid	795	32.85	1.940	0.002	−0.892	0.0097	0.0053
Isoheptadecanoic Acid	787	27.45	1.763	0.018	−0.925	0.0157	0.0083
Diglycerol	773	20.65	1.331	0.045	−0.916	0.0367	0.0195
Mannose-6-Phosphate	773	30.27	1.982	0.003	−0.758	0.0235	0.0139
2-Ketoisovaleric Acid	769	7.58	1.839	0.026	−1.340	0.0023	0.0009
Lauric Acid	762	18.62	1.646	0.019	−0.201	0.0026	0.0023
Arachidic Acid	758	31.27	1.695	0.017	−1.041	0.0172	0.0084
1-Hexadecanol	752	24.93	2.019	0.001	0.462	0.0095	0.0131
Xanthurenic Acid	745	29.3	1.931	0.022	−1.418	0.0043	0.0016
Shikimic Acid	688	21.65	1.441	0.050	5.448	0.0022	0.0963
1-Monostearin	680	33.89	1.625	0.018	−1.251	0.0140	0.0059
Monomyristin	679	30.81	1.728	0.027	−0.994	0.0038	0.0019
Vanillic Acid	671	21.06	1.890	0.005	−1.246	0.2311	0.0974
Alloxanoic Acid	664	29.12	1.576	0.017	4.139	0.0012	0.0220
Trisaccharide	656	35.35	1.841	0.022	−0.911	0.0065	0.0034
Urocanic Acid	637	25.85	1.780	0.018	−0.709	0.0035	0.0022
Spermine	616	33.04	1.838	0.020	−0.560	0.0060	0.0041
Glycyl Tyrosine	610	32.88	1.575	0.030	−0.923	0.0020	0.0010
Glutamyl-Valine	608	26.71	1.725	0.006	−1.210	0.0065	0.0028
Indole-3-Acetate	578	25.2	1.739	0.017	−1.010	0.0098	0.0049
Glucose D7 Labeled	538	23.82	1.710	0.011	−0.405	0.0030	0.0023
Acetaminophen	493	17.85	1.793	0.005	0.569	0.0031	0.0047
Tyramine	397	24.6	1.561	0.041	−0.256	0.0013	0.0011

### Principal component analysis

**Figure 2:**
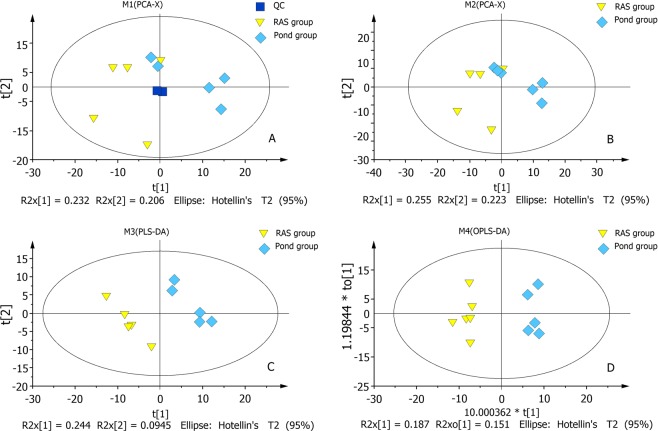
Score plot of PCA (principal component analysis) modeling for different treatments. (**A**) The PCA (principal component analysis) score plot of QC (Quality control), RAS and Pond groups in *Siniperca chuatsi* serum metabolites. (**B**) PCA score plots for pairwise comparisons between RAS and Pond group. (**C**) PLS-DA score plots for pairwise comparisons between RAS and Pond group. (**D**) OPLS-DA score plots for pairwise comparisons between RAS and Pond group.

**Figure 3:**
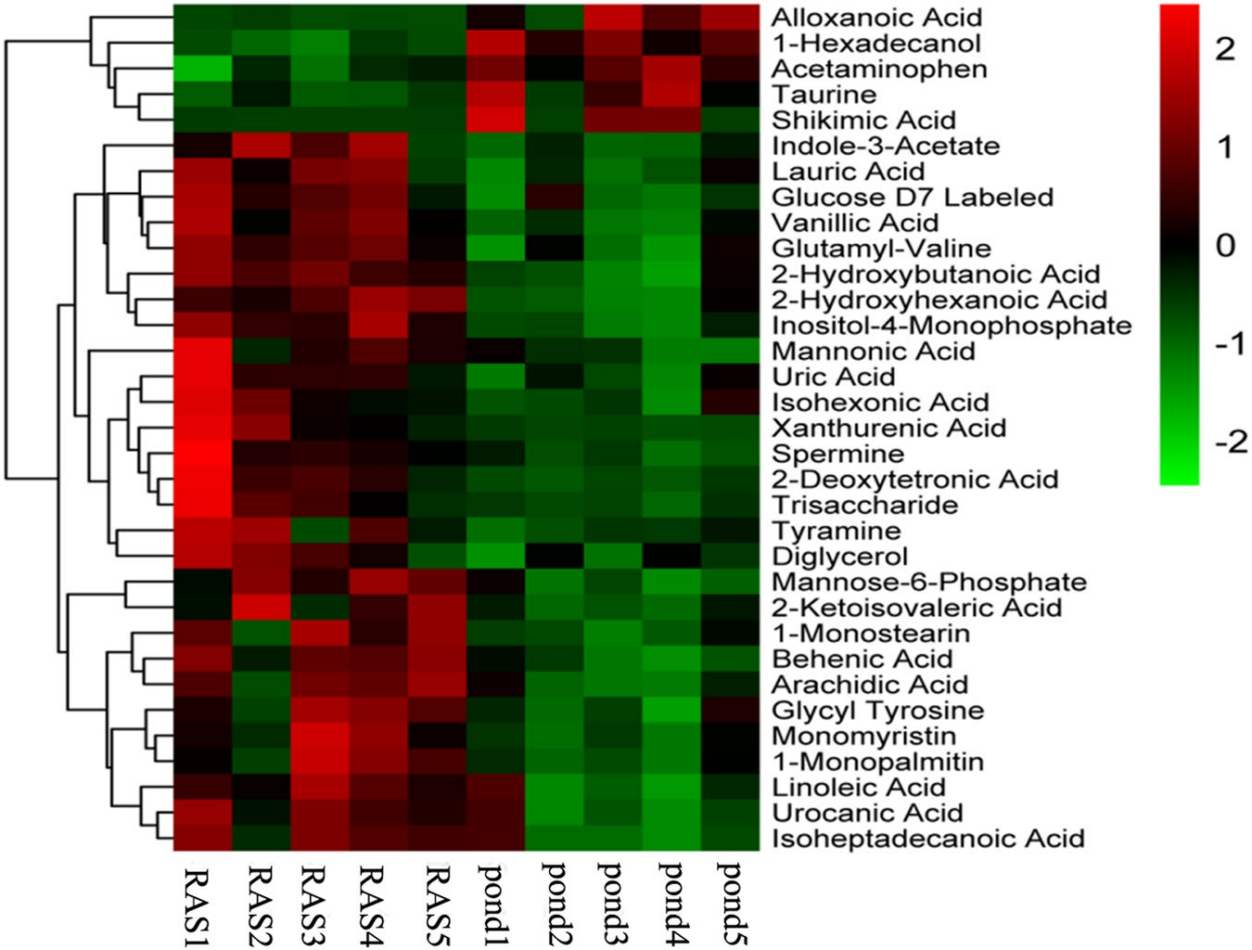
Heat map of varied abundance of metabolites. Red and green indicate increase and decrease of metabolites relative to the median metabolite level, respectively (see color scale).

**Figure 4:**
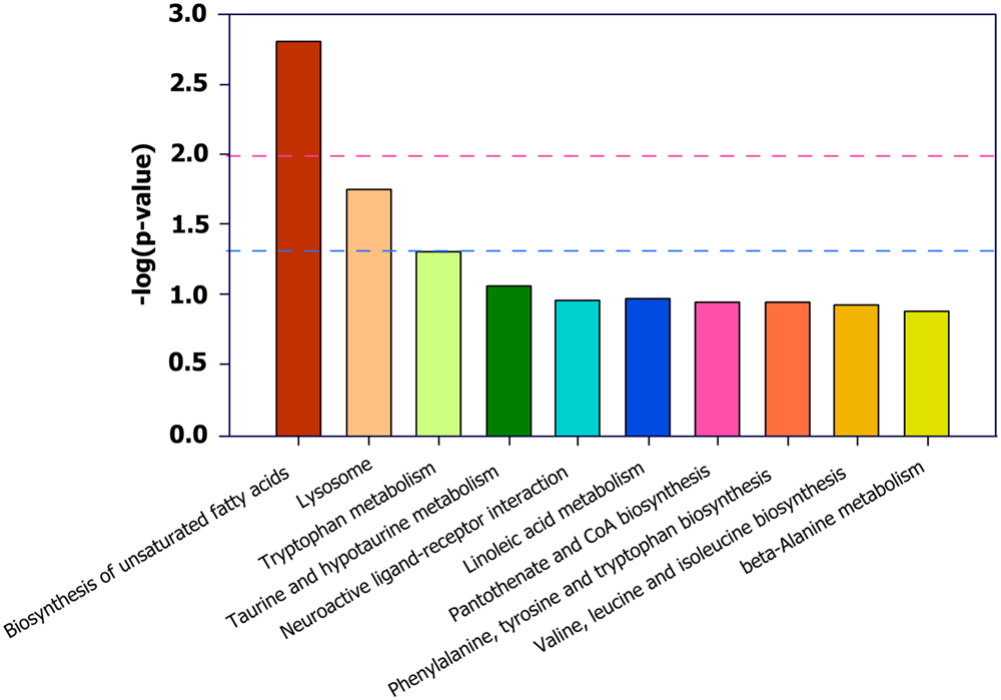
Kyoto encyclopedia of genes and genomes (KEGG) pathway enrichment analysis of *Siniperca chuatsi* serum differential metabolites in RAS and Pond groups. All the matched pathways are displayed in columns, and the length represents the fold change of enrichment, the pink dotted line represents the 0.01 P-value and the blue dotted line represents the 0.05 P-value. Total: the total number of metabolite in that set pathway; Expected = the expectation value; Hits = the number of metabolites in the experimental set matching the pathway set; Holm P = the P-value adjusted by Holm-Bonferroni method; FDR = the P-value adjusted using False Discovery Rate; Impact = the pathway impact value.

To reduce the complexity of the datasets, PCA was applied. The PCA score plot of QC (Quality control), RAS and Pond groups from *Siniperca chuatsi* serum metabolites was shown in Fig. 2A. PCA score plot indicated a clear difference in all metabolites between the pond and RAS groups (Fig. 2B). The *R*2*X* value of the PCA model was 0.255 in the *Siniperca chuatsi* serum. All samples from RAS and Pond groups fell outside the Hotelling’s *T*2 tolerance ellipse with 95% confidence, which indicated that no outlier was observed among the samples analysed. The validation plot for the PLS-DA model obviously revealed that the permutation tests of the serum were valid (*R*2*X* = 0.244, *R*2*Y* = 0.095) (Fig. 2C), which was the satisfactory effectiveness of the model. All the samples between the RAS and Pond groups were within the 95% Hotelling’s *T*2 ellipse based on the score plots of OPLS-DA model (Fig. 2D). As shown in Table 1, a total of 33 differential metabolites were identified between the RAS and pond groups (VIP > 1, *p* < 0.05), which was made of four amino acids and their derivatives, three saccharides, eleven organic acids, nine fatty acids and six other metabolites. Further, 33 identified metabolites revealed the existence of distinct differences between the RAS and pond groups by hierarchical cluster analysis (Fig. 3). Based on the clustering result of metabolites, serine, glycine, threonine, proline, asparagine, valine, glutamic acid, propanoic acid, lysine, hexadecanoic acid, octadecanoic acid and ornithine were abundant in each sample of the RAS groups, alloxanoic acid, 1-hexadecanol, acetaminophen, taurine, shikimic acid were abundant in each sample of the pond group. The KEGG pathway analysis was performed by MetaboAnalyst 3.0. Functional pathway analysis facilitating further biological interpretation revealed the most relevant pathways such as Biosynthesis of unsaturated fatty acids, Lysosome, Tryptophan metabolism, Taurine and hypotaurine metabolism, Neuroactive ligand-receptor interaction, Linoleic acid metabolism, Pantothenate and CoA biosynthesis; Phenylalanine, tyrosine and tryptophan biosynthesis; Valine, leucine and isoleucine biosynthesis; beta-Alanine metabolism, Propanoate metabolism, Glutathione metabolism, isoleucine degradation, leucine and Valine; Histidine metabolism, Mannose and fructose metabolism, Primary bile acid biosynthesis, Fatty acid biosynthesis, Fatty acid metabolism, Tyrosine metabolism, Nucleotide sugar and amino sugar metabolism, Proline and arginine metabolism, ABC transporters, Purine metabolism, Biosynthesis of secondary metabolites (Table S2). KEGG pathway mapper indicated that ten metabolic signaling pathways were significantly different among two groups (p < 0.05), including Biosynthesis of unsaturated fatty acids, Lysosome, Tryptophan metabolism, Taurine and hypotaurine metabolism, Neuroactive ligand-receptor interaction, Linoleic acid metabolism, Pantothenate and CoA biosynthesis, Tryptophan and tyrosine biosynthesis, Leucine, Phenylalanine, Isoleucine and Valine biosynthesis and beta-Alanine metabolism (Table S2). The three metabolic pathways of Biosynthesis of unsaturated fatty acids, Lysosome and Tryptophan metabolism were significant differences between the RAS and pond groups (Fig. 4).

## Discussion

The mandarin fish has a relatively high market value in China^3,15^, which is widely cultured throughout the country due to their rapid growth, large size, high nutritional value and delicious flesh and high price^3,15,16^. The successful artificial reproduction, fry and fingerling rearing technology of *S. chuatsi* could have potential for meeting the demands of *S. chuatsi* for commercial fish culture in China^1,17^. Mandarin fish is a key demersal piscivore in lakes and river systems, which feed on live shrimps, fish and other aquatic animals that are smaller than themselves, particularly fish. In the wild the fry of *S. chuatsi* start feeding, they prefer to take live prey fish and not take dead prey fish or artificial diets^18–20^. Recently, RAS have been well studied, and it has been proven major leaps in fish culture to maximize profit by increasing production, lowering costs and conserving water^21–23^. Therefore, in the present study, *S. chuatsi* were farmed in the RAS and pond, respectively, which were fed on live fry of cultivated *Hypophthalmichthys molitrix* and *Aristichthys nobilis*. In this study, the metabolite profiling was investigated using GC/MS to determine the metabolic characteristics of RAS and pond-cultured *S. chuatsi*, which could be regarded as the ultimate responses of biological systems to the environmental changes^24–26^. Up to now, many powerful analytical techniques have been used for identifying metabolite profiling, such as GC-MS and HPLC-MS (high performance liquid chromatography with mass spectrometry)^27–29^. GC-MS as a mature technology has a lot of advantages, such as good reproducibility, high sensitivity, high resolution, with a large standard library, relatively low cost and powerful analysis, which can be used for analyzing the primary metabolism products, including carbohydrates, organic acids, amino acids, and fatty acids^30–32^. For instance, glucose, oxypurinol, taurine, lactate, creatine, glutamate, alanine, sn-glycerol-3-phosphorylcholine, glycine, phenol, hypoxanthine, acetic acid, lysine, leucine and valine were main hepatopancreas metabolites differing in serum of normal grass carp based on ^1^H-NMR^33^. The metabolic differences were revealed in *Eriocheir sinensis* fed with dietary olive oil or palm oil using untargeted GC-MS metabolomics^31^. 68 metabolites were identified with high credibility and five metabolites were significantly different between PO group and OO group^31^. The derivatization is required in order to achieve prior to the analysis using GC-MS because of most of these metabolites are nonvolatile^34^.

Currently, we applied the GC-MS metabolomics approach revealed metabolic differences in the RAS- and pond-farmed *S. chuatsi* fed with dietary *Hypophthalmichthys molitrix* and *Aristichthys nobilis* because of the metabolites in circulation have been commonly employed to analyze the physiological status in response to multiple factors, including stresses, feed change, disease and so on^35^. So far, this is the first time to evaluate metabolic changes in the RAS-farmed *S. chuatsi*. We have discriminated the *Siniperca chuatsi* from RAS and pond in a PCA analysis of GC-MS metabolites. PCA is an unsupervised pattern recognition method, which was performed to examine the intrinsic variation in the dataset^36,37^. The greatest metabolic variance in our studies were related to fish culture environment and not caused from farmed *Siniperca chuatsi*. Since the *S. chuatsi* were farmed in recirculating ponds aquaculture system and pond, respectively. 33 metabolites were significantly different (p < 0.05 and VIP > 1) between RAS and pond groups in the *S. chuatsi*. Among all of them, organic acids accounted for approximately 60.6%, and most of which were related to fatty acid metabolism. These metabolites were comprised of 19 metabolites with the similarity over 700 and 14 metabolites with similarity under700. Some marker metabolites were worth investigating in the future. Compared with the RAS group, the levels of taurine, 1-hexadecanol, shikimic acid, alloxanoic acid and acetaminophen were higher in the pond group. In fish, taurine is mainly conjugated with choline to produce bovine bile sulfonate in the liver. In contrast to fish, bilirubin is conjugated with sugar (mainly glucuronic acid), and excreted in the bile in mammals^38^. Since taurine is involved in various functions, like exogenous substances, cell protection, neuro-modulation or neuro-transmission, detoxification of endogenous^39^. A large amount of taurine accumulated in the fish body, but its precise physiological significance is uncertain^38^. The contents of twenty-eight metabolites increased notably in the RAS group, including vanillic acid, inositol-4-monophosphate, mannonic acid, linoleic acid, isohexonic acid, 2-hydroxyhexanoic acid, 2-deoxytetronic acid, uric acid, 2-hydroxybutanoic acid, 1-monopalmitin, behenic acid, isoheptadecanoic acid, diglycerol, mannose-6-phosphate, 2-ketoisovaleric acid, lauric acid, arachidic acid and xanthurenic acid, 1-monostearin, monomyristin, trisaccharide, urocanic acid, spermine, glycyl tyrosine, glutamyl-valine, indole-3-acetate, glucose D7 labeled and tyramine. In this study, RAS has a lot of advantages, such as more dissolved oxygen and improving lots of environmental factors (TAN, NO^−^_2_-N, P and COD), which can provide comprehensive benefits to the *S. chuatsi*. The content of vanillic acid is the highest in the RAS group. Vanillic acid belonged to cinnamic acid derivative with strong antioxidant and antibacterial activity^40^. Inositol-phosphates, as a class of signal molecule, play an important role in cell signal transduction, and are related to a lot of physical activities. The mechanism of inositol phosphate signal transduction is closely related to the generation, metabolism and biological transformation of inositol phosphate. The nature is various chemical reactions (phosphorylation/dephosphorylation, interactions with inositol phosphate receptors). Mutual transformations of these reactions present a very complex network control system^41^. As compared to the pond group, Oleic acid and1-Monopalmitin in RAS group showed a clear increasing trend, which might be originated from the desaturation of saturated fatty acids^42^. Besides, other kinds of compounds, like amines, esters, alcohols and so on, were also identified in the RAS and pond groups, which could devote to the formation of the secondary metabolites that played a key role in the flavor components^43^. Significant changes in the levels of the main metabolites might show its key role in a number of metabolic pathways for regulating adaptation of the RAS- and pond-cultured *S. chuatsi*. The present study revealed that integration of metabolomics could be used to produce complementary data, which contribute to a better understand regarding correlation of fermentation process. However, there are still few limitations with metabolic differences of RAS- and pond-farmed *S. chuatsi*. The most obvious reason was only 335 identified metabolites in the RAS and pond groups, which might be attributed to high molecular weight metabolites and some volatile metabolites being missed. Thus, more sensitive high-throughput and advanced omics technologies should be used to accounts for the entire metabolic network. KEGG pathway mapper and metabolite set enrichment analysis suggested that these metabolites were related mainly to unsaturated fatty acid synthesis, Lysosome and amino acid metabolism.

In conclusion, this study was conducted to evaluate the use of GC-MS metabolomic platform to comprehensively analyze the effects of recirculating ponds aquaculture system (RAS) on the *S. chuatsi* metabolism. Database searching and statistical analysis revealed that there were altogether 335 metabolites quantified (similarity > 0) and 205 metabolites were described by mass spectrum matching with the similarity over 700. Among the total 335 metabolites quantified, 33 metabolites were significantly different (p < 0.05and VIP > 1) between RAS and pond groups. Biosynthesis of unsaturated fatty acids, lysosome, tryptophan metabolism were recommended as the KEGG pathway maps for *S. chuatsi* farmed in RAS. The present study revealed that RAS could provide comprehensive benefits to the effects of *S. chuatsi* metabolism, which suggested RAS was an efficient, economic, and environmentally friendly farming system compared to pond system.

## Methods

### Ethics statement

Investigations and protocols were conducted according to the guiding principles for the use and care of laboratory animals and in compliance with Anhui Science and Technology University Institute of Animal Care and Use Committee. The institutional review board approved this procedure. Our study had been submitted to and approved by the Academic Ethics Committee of Anhui Science and Technology University. All sample collection was undertaken in accordance with relevant Academic Ethics Committee of Anhui Science and Technology University guidelines and regulations.

### Fish housing and feeding

*S. chuatsi* juveniles were farmed in the RAS and pond of Chuzhou Nanqiao District Yangtze River Aquaculture Breeding Ground (Chuzhou, China) from July to September 2018. The fish were cultivated with a 300 m^3^ running water aquaculture pond (30 × 5 × 2 m) and 4200 m^3^ pond (60 × 35 × 2 m). The culturing density in RAS and pond were 180.6 g /m^3^ and 8.0 g /m^3^, respectively. The dissolved oxygen (DO), ammonia nitrogen (NH_4_-N), pH, nitrite content (NC), etc., were monitored in two RAS and pond of *S. chuatsi*. The RAS and pond of water quality indicators were DO 7.50 ± 0.29 mg/ L, 6.48 ± 0.38 mg/ L, 0.0016 ± 0.0007 mg/ L, 0.0021 ± 0.0009 mg/ L, pH 7.33 ± 0.41, 7.09 ± 0.51, NC 0.048 ± 0.014 mg/ L, 0.198 ± 0.065 mg/ L, respectively. The perch were provided a live prey fish (*Hypophthalmichthys molitrix* and *Aristichthys nobilis*) during 3- month experiments. The mandarin fish reached average terminal body length of 28.98 ± 0.65 cm and 18.64 ± 0.83 cm, average terminal body weight of 426.68 ± 60.85 g and 135.6 ± 27.85 g after 90 days rearing from average initial body length of 7.12 ± 0.53 cm, average initial body weight of 2.58 ± 1.15 g, respectively. Five samples of *S. chuatsi* were caught by brail fishing net in the RAS and pond, respectively. All captured fish were transferred to the laboratory maintaining a continuous supply of oxygenated water in refrigerated van for 1.5 h at 25 °C. Each fish was anesthetized by tricaine methanesulfonate (MS-222, 0.2 g/L) for 4 min, and blood was obtained by venipuncture from the tail vein of *S. chuatsi*, and placed in serum-separating tubes. The blood of Mandarin fish was allowed to clot at room temperature for 30 min. Serum was obtained by centrifugation (10 min, 2000 rpm at 4 °C), and stored frozen at −80 °C.

### Sample preparation

To evaluate the metabolism status, serum metabonomic profiles of RAS- and pond-cultured *S. chuatsi* were detected using GC-MS techniques employed to globally characterize changes in this study. Ten serum samples were slowly thawed at room temperature for calibration curves and quality control (QC) samples. 20 μL internal standard (2-chloro-l-phenylalanine, 0.3 mg/mL) and 600 μL extraction solvent with methanol/water (4/1, v/v) were added to each sample. Samples were stored at −80 °C for 2 min and then grinded at 60 HZ for 2 min. 120 μL of chloroform was added to the samples, then the samples were vigorously vortexed and followed by 10 min ultrasound-associated extraction at ambient temperature, then stored at 4 °C (10 min). The samples were centrifuged at 12000 rpm for 10 min at 4 °C. QC sample was prepared by mixing aliquots of the all samples to be a pooled sample. An aliquot of the 150 μL supernatant was transferred to a glass sampling vial for vacuum-dry at room temperature. And 80 μL of methoxylamine hydrochloride (dissolved in pyridine, 15 mg/mL) was subsequently added. The resultant mixture was vortexed vigorously for 2 min and incubated at 37 °C for 90 min. 80 μL of BSTFA (with 1% TMCS) and 20 μL n-hexane were added into the mixture, which was vortexed vigorously for 2 min and then derivatized at 70 °C for 60 min. The samples were allowed to placed at ambient temperature for 30 min before GC-MS analysis.

### GC–MS analysis

After the completion of sample pretreatment, the samples were sent to Shanghai OE Biotech. Co., Ltd. (Shanghai, China) for GC-MS analysis. Data were log2 transformed by using Microsoft Excel, and the resulting data matrix was then imported into SIMCA-P software (version 14.0, Umetrics, Umea, Sweden). Principle component analysis (PCA) and (orthogonal) partial least-squares-discriminant analysis (O) PLS-DA^31^ were used to evaluate the metabolic difference among RAS and pond groups, after mean centering and unit variance scaling. The ellipse represents the Hotelling’s T2 with 95% confidence interval of the modeled variation^44^. The heat map was produced in the R environment for statistical computing. The heat map color drawn using R with ggplot2 represents the z-score transformed raw data for RAS-farmed *S. chuatsi* metabolites^45^. Variable importance in the projection (VIP) statistics from OPLS-DA modeling was used to identify the overall contribution of each variable. Those variables (*p* < 0.05, VIP >1.0) are considered relevant for group discrimination. Furthermore, the proteins were further identified according to Kyoto Encyclopedia of Genes and Genomes (KEGG, http://www.genome.jp/kegg/)^32^.

## Supplementary information

Table S1.

Table S2.
